# Supplementary material for: Avoidance of non-localizable obstacles in echolocating bats: A robotic model
Source: PLoS Comput Biol. 2019 Dec 19;15(12):e1007550. doi: 10.1371/journal.pcbi.1007550 (PMC6941896; doi:10.1371/journal.pcbi.1007550)
Supplement: Attachment — Submitted filename: Revision Plos comp Bio.pdf [file pcbi.1007550.s001.pdf]

We wish to thank both reviewers for their encouragement and helpful remarks. We thank the reviewers for their input in the acknowledgment section of the paper.

Reviewer 1

It is not clear if the purpose of the work is to provide robotics with novel obstacle-avoidance techniques, or if the robot platform is used to test the biological hypothesis. I assume the latter, otherwise the lack of comparison with other obstacle avoidance techniques would make the paper quite weak.

Indeed, our paper tests a biological hypothesis. We do not wish to make claims about the presented methods being a competitive method for obstacle avoidance in robots.

The conclusions are based on a limited number of experiments (5 trials in the rectangular arena, 15 in the corridor) so their statistical significance is limited, I think that more experiments would make the paper much stronger.

It is true that this number of experimental trials was limited, but it should be appreciated that each trial contains a high number of potential collision events to test the obstacle avoidance behaviour. Nevertheless, we agree that more trials would reduce uncertainty about the results. Hence, we have now doubled the amount of data collected. As can be seen, the experimental results did not change markedly. However, as expected, the confidence intervals around the averages decreased.

Regarding the comparison of fixed/scanning gaze, I think that this is a bit unfair since the poorer behaviour of the latter may be influenced by the speed of the scanning w.r.t. the robot's speed (both linear and angular) and manoeuvring capabilities. So faster scanning and/or lower robot speed may produce less collisions (and vice-versa). This aspect should be considered and -- if possible- assessed experimentally.

We agree with the reviewer that faster scanning in combination with a lower robot speed would increase the performance. However, as we are testing a hypothesis about sonar behavior in bats, we are bound by the parameters that describe their behavior. The ‘flight’ speed, rotational velocity and scanning speed have all been parameterized based on data describing observed bat behavior.

However, the reviewer’s comment made us think about the behavior of certain subgroups of bats. Some bats (called active gleaners) forage among dense vegetation, these include nectarivorous and insectivorous bats. At least part of their flight is spent hovering. The very low flight speed of these bats might open up an opportunity for more scanning behavior (which they do while homing in on prey or flowers).

Please describe better which is the purpose of the random reflectors and of the egg cartoons in the corridor.

The corridor experiments were included to mimic the experimental settings in which bats have been tested before. For example, Warnecke et al. (2016, 2018, 2018a,) and Knowles (2015) flew bats through corridors lined with poles. The behavioral and neurophysiological results of these studies suggest that poles spaced by less than 15 cm are not perceived as separate obstacles by the bats. Rather, in the words of Warnecke et al 2018, responses to echo cascades from the densely spaced poles represent a single extended stimulus event that lasts over 35–40 ms. This implies that, under these conditions, the bat is not following the corridor by localizing the obstacles and planning a path between them. The sensorimotor loop underlying the behavior in these experiments must rely on other cues. We hypothesise that IID might be a sensible cue.

In our experiments, we wanted to present the robot with stochastic reflectors that present a worst case scenario for a theory based on localization of reflectors: the egg cartons return many, overlapping echoes which can not be individually localized in space. Nevertheless, our robot, using an IID based strategy can follow the corridor.

In brief then, the egg cartons were used to ascertain that the environment presented a condition under which bats would not be able to use localization cues, showing that our hypothesized algorithm works robustly whether reflectors can be individually localized or not. We have made this point more explicitly in the introduction.

Fig 1 is too small.

We have changed the layout of the figure to expand some of the panels.

Reviewer 2

This is an excellent combination analysis based on behavioral performance of echolocating bats and testing of a robotic model of obstacle avoidance that compares two different strategies for aiming the model bat's head and sonar beam. In one model the beam is aimed straight ahead in the upcoming path, not side-to-side, so that the obstacles that surround the path are kept to the sides, leaving the upcoming path to be checked for whether it is open or obstructed. Behavioral data that support this method are described in a paper by Knowles, at al., 2015. In a cluttered scene involving choices about which potential upcoming path is unobstructed, the bat aims its beam into the path, turning slightly to anticipate and imminent steering action. It does not scan left and right. A paper (Temporal binding of neural responses for focused attention in biosonar. James A. Simmons Journal of Experimental Biology 2014 217: 2834-2843; doi: 10.1242/jeb.104380) examines the underlying perceptual mechanism. The alternative

method, scanning left and right, is typical of bats flying in open, uncluttered spaces while searching for insects. This is well-documented in the manuscript's references. One point worth mentioning is that the robotic model assumes that bats determine the horizontal direction of an object from the binaural difference in echo amplitude. The evidence suggests that the bat uses binaural echo delay differences and that the amplitude difference serves to magnify the time difference through the process of amplitude-latency trading, as described in the Pollak reference in the manuscript. These details need to be given in the manuscript because they strengthen the authors' case.

We fully agree with the reviewer that both time and level difference cues are available to the bat to steer its gaze. We have restricted ourselves to ILD cues for pragmatic reasons only. For our simple system, the ITD cues would be dominated by the ILD cues when transformed by time-intensity trading into an exclusively time based internal representation. Furthermore, we could not find sufficiently detailed experimental data on this time-intensity trading mechanism to allow us to properly model this transformation for the entire range of echo intensity values encountered in our experiments. We have included the text below in the discussion to make this point more clearly.

"In the echolocation system both interaural level differences and interaural time differences provide angular information. Given the small size of the bat's head and time-intensity trading occurring in the neural responses to received echoes it has been argued that the most robust source of angular information available to bats is the interaural level difference (Pollak, 1988). On the other hand, when confronted with complex echo signals bats possess neural populations that code for interaural time differences of the envelopes of these complex echoes (Borina, 2011). The acoustic attention scheme proposed by Simmons [JEB2014, Temporal binding of neural responses for focused attention in biosonar. James A. Simmons Journal of Experimental Biology 2014 217: 2834-2843; doi: 10.1242/jeb.104380] describes how time and intensity cues can be consistently combined, whereby level differences amplify, through time-intensity trading, the physically occurring time differences. However, in our simple controller, implementing the same time-intensity trading mechanism would result in the smaller interaural time difference cues being dominated by the interaural level difference cues. Hence, as both cues are highly correlated, we have chosen to make use only of the relative loudness of echoes in the left and the right ear as a robust (although, not perfect) indicator about whether the reflectors are more likely to be located left or right from the midline."
